# Comparative Transcriptome Analysis of Milk Somatic Cells During Lactation Between Two Intensively Reared Dairy Sheep Breeds

**DOI:** 10.3389/fgene.2021.700489

**Published:** 2021-07-19

**Authors:** Sofia Michailidou, Athanasios Gelasakis, Georgios Banos, George Arsenos, Anagnostis Argiriou

**Affiliations:** ^1^Institute of Applied Biosciences, Center for Research and Technology Hellas, Thessaloniki, Greece; ^2^Laboratory of Animal Husbandry, Faculty of Health Sciences, School of Veterinary Medicine, Aristotle University of Thessaloniki, Thessaloniki, Greece; ^3^Laboratory of Anatomy and Physiology of Farm Animals, Department of Animal Science, School of Animal Biosciences, Agricultural University of Athens, Athens, Greece; ^4^Scotland’s Rural College, Easter Bush, Edinburgh, United Kingdom; ^5^Department of Food Science and Nutrition, University of the Aegean, Lemnos, Greece

**Keywords:** Chios breed, Lacaune breed, mammary gland, transcriptome sequencing, differential expression, milk production, breeding strategies, milk somatic cells

## Abstract

In dairy sheep industry, milk production dictates the value of a ewe. Milk production is directly related to the morphology and physiology of the mammary gland; both being designated targets of breeding strategies. Although within a flock breeding parameters are mutual, large differences in milk production among individual ewes are usually observed. In this work, we tested two of the most productive dairy sheep breeds reared intensively in Greece, one local the Chios breed and one foreign the Lacaune breed. We used transcriptome sequencing to reveal molecular mechanisms that render the mammary gland highly productive or not. While highly expressed genes (caseins and major whey protein genes) were common among breeds, differences were observed in differentially expressed genes. *ENSOARG00000008077*, as a member of ribosomal protein 14 family, together with *LPCAT2*, *CCR3*, *GPSM2*, *ZNF131*, and *ASIP* were among the genes significantly differentiating mammary gland’s productivity in high yielding ewes. Gene ontology terms were mainly linked to the inherent transcriptional activity of the mammary gland (GO:0005524, GO:0030552, GO:0016740, GO:0004842), lipid transfer activity (GO:0005319) and innate immunity (GO:0002376, GO:0075528, GO:0002520). In addition, clusters of genes affecting zinc and iron trafficking into mitochondria were highlighted for high yielding ewes (GO:0071294, GO:0010043). Our analyses provide insights into the molecular pathways involved in lactation between ewes of different performances. Results revealed management issues that should be addressed by breeders in order to move toward increased milk yields through selection of the desired phenotypes. Our results will also contribute toward the selection of the most resilient and productive ewes, thus, will strengthen the existing breeding systems against a spectrum of environmental threats.

## Introduction

In the Mediterranean basin, dairy sheep have been milked since time immemorial to produce special dairy products. Milk production in dairy sheep is a metric that defines the value of individual animals and breeds in terms of economic profit and farm sustainability. Even for meat sheep breeds, lactation performance is important during the suckling period, to accelerate lamb growth until weaning age. Particularly in dairy sheep, breeders were traditionally selecting ewes and design breeding strategies based on milk yields, a practice that still applies in most dairy farms. This resulted, over time, in the dominance of certain dairy breeds such as Lacaune, Assaf, Awassi, and Friesian in flocks worldwide. However, with the advent of intensified dairy sheep farming systems, important traits such as resilience and adaptability to local environments were neglected.

In sheep, many studies focus on sequencing the transcriptome of muscles (Zhang et al., 2014), adipose tissues (Wang et al., 2014), or skin (Yao et al., 2019) and results are exploited as candidate markers in breeding schemes, toward the desired phenotype. Recently though, transcriptome sequencing in sheep targets mainly the mammary gland and in particular, the discovery of highly or differentially expressed genes among breeds or time-points of lactation (Paten et al., 2015; Suárez-Vega et al., 2015, 2017a; Hao et al., 2019; Farhadian et al., 2020; Wang et al., 2020). Although these studies have explored the expression profile of sheep’s mammary gland during different stages of lactation, little is known about the underlying mechanisms that render the mammary gland highly productive or not within breed in terms of milk yield. To this end, research on the expression profiles of long non-coding RNAs (lncRNAs) (Hao et al., 2020a) and circular RNA (circRNA) (Hao et al., 2020b) in the mammary gland of ewes of different performances has been conducted, investigating the interactions within the lncRNAs-mRNA and circRNA-miRNA networks, respectively, in relation to milk synthesis and mammary gland development.

In farm animals, productivity is shaped by single nucleotide polymorphisms (SNPs) and differences on gene expression levels, therefore, the direct comparison of transcripts is pursued between different animals, tissues and time-points in order to reveal where and when genes are active and the way these differences impact on desired traits. The notion is that differential gene expression is the ultimate approach in elucidating a cell’s function or a gland’s productivity in a given time point, thus, a variety of different software and pipelines have been developed to extract relative information (Costa-Silva et al., 2017). So far, 3,355 QTLs have been identified in the sheep genome, which impact 270 different traits (Hu H. et al., 2016). However, this number is expected to increase since transcriptome sequencing provides additional data on new transcripts and associations with traits that were until recently unknown.

Greek breeds of dairy sheep are characterized by high levels of genetic heterogeneity (Michailidou et al., 2018) and this is reflected in each ewe’s individual performance. Milk productivity is the main criterion for breed selection, especially in intensive systems of production that require considerable capital investment and their profitability depends directly to ewe’s productivity (Gelasakis et al., 2012). In Greece, the majority of intensively reared flocks of dairy sheep comprise Chios and Lacaune breeds. The Chios breed is the most productive indigenous dairy sheep breed, producing on average 307 kg of milk in 170 days (Basdagianni et al., 2019). The Lacaune breed is a foreign breed imported from France (Roquefort area); its performance has been exponentially improved following more than fifty years of genetic selection and improvement (Barillet et al., 2001). Based on official milk recording of 2014, the average milk production of Lacaune ewes is 302 kg in 168 days (Institute de l’Elevage, 2014). Lacaune sheep are well-adapted in Greece and demonstrate high lactation persistency. It is common, however, to observe ewes of the same breed reared in the same flock with extreme differences in milk yield.

Proper evaluation of genetic merit of an animal is desirable to identify the most productive individuals, to uncover resistance or susceptibility against diseases and ultimately to define breeding strategies, thus design the reproductive management of flocks. Greece, according to the 2019 Global Climate Risk Index (Eckstein et al., 2021) is ranked among the top countries affected by the impacts of climate change. The country is vulnerable to drought and rising temperatures (Demertzis and Iliadis, 2018) and pastures will be reduced in the upcoming years. Thus, selection for resilient and more productive animals is the only option to cope with future threats.

In the present study, we characterize and compare the transcriptional activity of the mammary gland in the two most intensively reared dairy sheep breeds in Greece (Chios and Lacaune). We compare mammary gland’s gene expression activity between ewes of extremely different performance (high and low yielding ewes) within and between breeds. The aim is to reveal the biological mechanisms that support milk production and to explore whether such clusters of genes can be used as potential markers to improve performance and facilitate selective breeding strategies.

## Materials and Methods

### Animals and Milk Sampling

Chios and Lacaune ewes were bred in two farms located in the regions of Thessaly and Central Macedonia, respectively. Details of farm locations and environmental factors are presented in Supplementary Table 1. In total, six non-related ewes from each breed were selected based on their pedigree data and available performance records. All ewes were intensively reared, fed with the same ration within breed and had *ad libitum* water access. Milk sampling was conducted during the same season for both breeds (June 2018) while all ewes were in their 2nd-3rd lactation period. Sampling for Chios breed (CH) was conducted when ewes were in day 120 post-partum, whereas for Lacaune breed (LA), ewes were in day 150 post-partum. These time-points correspond close to the end of normalized lactation for each breed, hence, possible biases in transcriptional activity due to different physiological stages of the mammary gland are eliminated. For each breed two groups (*n* = 3) were formed: one consisting of three high yielding ewes (HY) and another consisting of three low yielding (LY) ewes, in terms of milk production. All ewes were milked three times a day; milk sampling was performed during the morning milking. Each ewe was examined by an experienced veterinarian to assess health and welfare status including, body condition score (BCS) and udder health clinical assessment. California mastitis test (CMT) was applied before each sampling to ensure the absence of subclinical mastitis. On average, daily milk yields of selected ewes for the sampling period were 3.10 and 2.80 kg for CH-HY and LA-HY groups, respectively, whereas the respective numbers for the LY groups were 1.04 and 1.70 kg. Approximately 200 ml raw milk were collected in 4 × 50 ml sterile tubes, after discarding the first streams of milk. Milk samples were stored at 4°C, until further assayed in the laboratory.

### RNA Extraction and Library Construction

Total RNA was extracted from milk somatic cells; 150 ml of fresh milk were centrifuged at 600 × g for 10 min at 4°C, to pellet down -to the max- only eukaryotic and not bacterial cells. Total RNA was extracted from the cell pellet using the TRIzol^TM^ Reagent (Thermo Fisher Scientific, Waltham, MA, United States), following the standard protocol. Total RNA was quantified on a Qubit 4 using the Qubit^TM^ RNA BR Assay Kit (Thermo Fisher Scientific), and its integrity (presence of intact 28S and 18S rRNA subunits) was evaluated on an agarose 0.8% TAE gel.

Three micrograms of total RNA were used to isolate mRNA, using the NEBNext^®^ Poly(A) mRNA Magnetic Isolation Module (NewEngland BioLabs Inc.); mRNA libraries were further constructed using the NEBNext^®^ Ultra^TM^ RNA Library Prep Kit for Illumina^®^ (NewEngland BioLabs Inc.), according to the manufacturer’s instructions.

### Library Quantification and RNA Sequencing

All libraries were initially quantified with the Qubit 4 fluorometer using the Qubit^TM^ dsDNA BR Assay Kit (Thermo Fisher Scientific). Quality assessment and estimation of each library’s average size was evaluated on the Fragment Analyzer system (Agilent Technologies Inc. Santa Clara, United States), using the DNF-477-0500 kit. To further assess the molarity of each library, quantitative PCR (qPCR) was conducted on a Rotor-Gene Q thermocycler (Qiagen, Hilden, Germany) with the KAPA Library Quantification kit for Illumina sequencing platforms (KAPA BIOSYSTEMS, Woburn, MA, United States). Transcriptome sequencing was performed on a NextSeq500 system (Illumina, San Diego), with a NextSeq 500/550 High Output v2 kit (2 × 75 cycles), according to the manufacturer’s instructions.

### Bioinformatic Analysis

#### Quality Control and Alignment Rate

Raw reads were evaluated and quality trimmed from adaptors and low-quality sequences using a local installation of the Trim Galore wrapper version 0.4.1 (Krueger, 2015) with default parameters. To ensure sequences originated from the *Ovis aries* genome and not from other contaminants, quality trimmed reads were aligned to the sheep genome (Oar_v3.1) using the HISAT2 aligner version 2.0.5 (Kim et al., 2015). In addition, we used the *bowtie2* (Langmead and Salzberg, 2012) algorithm to align reads against ASM584v2 genome (RefSeq assembly accession: GCF_000005845.2), to test the presence of *E. coli* contaminants.

#### Sequence Alignment and Transcript Assembly

Transcript abundances and differential analyses were carried out using the “new Tuxedo” protocol (Pertea et al., 2016). Gene expression levels were calculated using the FPKM (Fragments Per Kilobase of gene per Million) normalization method. Gene annotation was conducted using index Oar_v3.1.98 as reference^1^. Briefly, sequences were aligned to the reference genome using the *–hisat2* command and assembled into potential transcripts using the *StringTie* assembler version 1.3.3 (Pertea et al., 2015). All assembled transcripts were further merged in a consolidated annotation set with –*merge* command for comparison purposes between samples and re-estimation of transcript abundances in subsequent steps. All analyses were implemented on a Linux/based HPC cluster assigning one node with 32 cores and 256 GB RAM.

#### Differential Expression Analysis

Differential expression (DE) analysis was carried out in R version 3.6.2 (R Development Core Team, 2011) with the Ballgown R package (Frazee et al., 2015). Comparisons were conducted within breeds (CH-HY vs. CH-LY; LA-HY vs. LA-LY) as well as between breeds (CH-HY vs. LA-HY; CH-LY vs. LA-LY). The level of differentiation among groups (fold change, fc) was estimated with the *stattest()* command in R. To exclude false positive estimations low-abundance transcripts were filtered out by removing all transcripts with a variance across all samples less than one. Within breed (Chios, Lacaune) or group (HY, LY) differentially expressed genes (DEGs) were calculated using the FPKM values and transcripts with a sum of FPKM value less than one were further removed. Significantly differentially expressed genes were acquired from DEGs, after filtering out those with *P*-value > 0.05. Of the remaining genes, those with a fourfold change that are up or down regulated within each breed or group are presented and discussed. All plots were created in R (R Development Core Team, 2011) and visualized by combining functions provided by the ggplot2 (Wickham, 2016), gplots (Warnes et al., 2019), EnhancedVolcano (Blighe et al., 2019), and pheatmap (Kolde, 2012) R packages.

#### Functional Analysis of Differentially Expressed Genes

Differentially expressed genes (fc > 4 and *P* < 0.05) were used for gene set enrichment analysis, with a threshold for false discovery rate (FDR) less than 0.05. Gene ontology and KEGG pathway enrichment analysis of DEGs were assessed using ShinyGO v0.61 (Ge et al., 2020), against sheep genome Oar_v3.1. DEGs for each group were categorized into three major functional types: biological processes, cellular components and molecular functions. For each functional classification, the 20 most significant terms are presented, where available.

## Results and Discussion

### Milk Somatic Cells for Mammary Gland Transcriptome Sequencing

Several sampling methods have been proposed for transcriptome sequencing of ruminant’s mammary gland, since the udder is a complex and heterogeneous gland that is comprised of multiple cell lineages (luminal, alveolar, and myoepithelial cells) (Van Keymeulen et al., 2011; Cánovas et al., 2014). Certain methods such as sampling after killing cows by electroshock (Cui et al., 2014) or by using needle biopsies (Paten et al., 2015) are more precise in cell sampling but are also invasive (Yang et al., 2016) and do not respect animal welfare rules, whereas in the case of biopsies lactation is interrupted by the obligatory use of antibiotics. On the other hand, the unspecific tissue sampling complicates the ensuing bioinformatics analysis and increases costs since many sequences are omitted. To avoid such problems, we sequenced the mammary gland transcriptome using RNA extracted from milk somatic cells (MSCs). This method is easier to apply, since sampling is performed during the routine daily milking and it is easy to study groups or individual animals at different time-points (Suárez-Vega et al., 2015). Moreover, research has shown that MSCs constitutes a representative RNA source of the mammary gland (Medrano et al., 2010; Cánovas et al., 2014). Alternatively, RNA sequencing from mammary gland can be performed using milk fat globules, which enclose mammary epithelial cells, however, low molecular weight RNA is obtained in such a case (especially for the 18S and 28S rRNA) (Cánovas et al., 2014). In addition, the g-force during centrifugation is crucial since at higher forces bacterial cells are pelleted down due to their size. Suárez-Vega et al. (2015) showed that milk centrifugation at 540 x g did not extract, therefore sequence any *Escherichia coli* reads, in contrast to a study by Cánovas et al. (2014) which showed that milk centrifugation at 2,000 × g led to the production of million sequences of bacterial origin, with *E. coli* being the dominant species. In our dataset however, alignment of reads against ASM584v2 genome did not reveal the presence of *E. coli* reads. Concerning the appropriate amount of milk for RNA extraction, several volumes are proposed, with 150 ml being the most suitable in terms of yield and quality (Mura et al., 2013). In our analysis, centrifugation of 150 ml milk at 600 × g resulted on average in 3–5 μg of high-quality total RNA per sample.

### RNA-Seq Data and Summary Statistics

Raw and quality filtered reads for each sample are presented in Supplementary Table 2. In total 311,937,466 raw reads were produced, corresponding approximately to 26 M reads per sample. Raw sequences have been deposited to NCBI Sequence Read Archive (SRA) under the BioProject PRJNA724691 and Biosample accession numbers SAMN18848576–SAMN18848587. HISAT2 aligner revealed a high alignment rate for some samples and intermediate for others, indicating possible presence of contaminants, other than *E. coli*. The depth of sequencing was designed considering all economic costs and benefits together with the ability to detect low abundant transcripts. To this end, several tools have been developed suggesting the optimal number of biological replicates and sequencing depth for differential expression experiments (Łabaj et al., 2011; Busby et al., 2013; Hart et al., 2013; Zhao et al., 2018). We assessed the correlation of samples per breed through pairwise comparisons of the Pearson correlation coefficient (*r*^2^). Mean *r*^2^-values for Chios and Lacaune breeds were 0.894 and 0.697, respectively (Supplementary Table 3). Although for technical replicates Spearman’s rank correlation coefficient should exceed 0.9 (Mortazavi et al., 2008), for biological replicates there is not an acceptable threshold set either for Pearson or Spearman correlation coefficients, since many factors are involved, thus can increase sample heterogeneity. Mean *r*^2^-values for Lacaune breed were particularly low when LA7-H was included in pairwise comparisons, pointing to a differentiated transcriptional activity of its mammary gland, although the available phenotypic data pointed otherwise.

Overall, 74,274 transcripts were assembled; of these 44,050 transcripts remained after filtering out low-abundance transcripts. The longest transcript was 41.063 kb, belonging to the *choline kinase alpha* (*CHKA*) gene, with a mean and median length among all transcripts of 2,519 and 1,665 bp, respectively (Supplementary Figure 1). As presented in Table 1, the majority of identified transcripts were of low abundance (mean of FPKM in biological replicates per group < 10). The number of highly expressed transcripts ranged from 87 (LA-LY) to 109 (CH-LY), and in all cases transcripts encoding for caseins and whey proteins were among them. Removal of low abundant transcripts, although in many cases at arbitrary thresholds, normalizes errors introduced during sampling, experimental library construction or sequencing, and increases the power of analysis (Risso et al., 2011; Dillies et al., 2013; Su et al., 2014). In our dataset, many transcripts corresponded to the same gene, thus, 27,327 unique genes (21,566 for Chios and 27,292 for Lacaune breeds) were identified (Supplementary Figure 2), including coding and non-coding genes. The number of expressed genes varies and is affected by the production stage of the ewe during sampling. For instance, the number of expressed genes in MSCs in Holstein cows are reportedly 16,892 during the onset of lactation, increase to 19,094 during peak lactation and then decrease to 18,070 during the final stages of lactation (Wickramasinghe et al., 2012). Given the different collection time-points for Chios and Lacaune breeds, deviation in the number of expressed genes could be attributed to the mammary gland’s productive activity, although these time-points are close to the end of normalized lactation period for each breed. For Assaf and Churra breeds, Suárez-Vega et al. (2015) identified 16,219 and 16,747 genes for day 120 and 16,404 and 16,850 genes for day 150, respectively. Paten et al. (2015) identified 10,132 and 10,096 expressed genes in the mammary gland of Romney ewes during the last stages of gestation and at the beginning of lactation, respectively. The reduced number of expressed genes compared to our dataset, apart from the differences in breeds and sampling time-point, could be attributed to the use of an updated reference genome release (Oar_v3.1.98 version) in which the number of predicted or uncharacterized gene transcripts is reduced. Also, Paten et al. (2015) used a needle biopsy during sampling in which, apart from MSCs, myoepithelial, immune and stromal cells are present (Cánovas et al., 2014). Among the studied breeds a high percentage of common genes was observed (78.8% corresponding to 21,533 genes); 35 and 5,761 genes were exclusively expressed in Chios and Lacaune breeds, respectively, suggesting a large impact of breed on mammary gland transcriptome activity. Regarding the total number of expressed genes per group, 27,294 and 22,435 genes were identified for HY and LY groups, respectively. Numbers of common, shared and unique genes among all groups are presented in Supplementary Figure 3.

**TABLE 1:** Classification of transcript abundance based on FPKM (Fragments Per Kilobase of gene per Million) values, per breed and production group.

Breed	Chios		Lacaune	
Group (High yield/Low yield)	HY	LY	HY	LY
**Number of highly expressed transcripts (FPKM ≥ 500)**	94	109	103	87
**No. moderate expressed transcripts (10 ≤ FPKM < 500)**	4,243	4,713	4,379	4,441
**No. lowly expressed transcripts (FPKM < 10)**	32,057	34,961	37,349	34,777
**No. non-expressed transcripts**	7,656	4,267	2,219	4,745
**Total number of transcripts identified**	44,050	44,050	44,050	44,050

**FIGURE 1:**
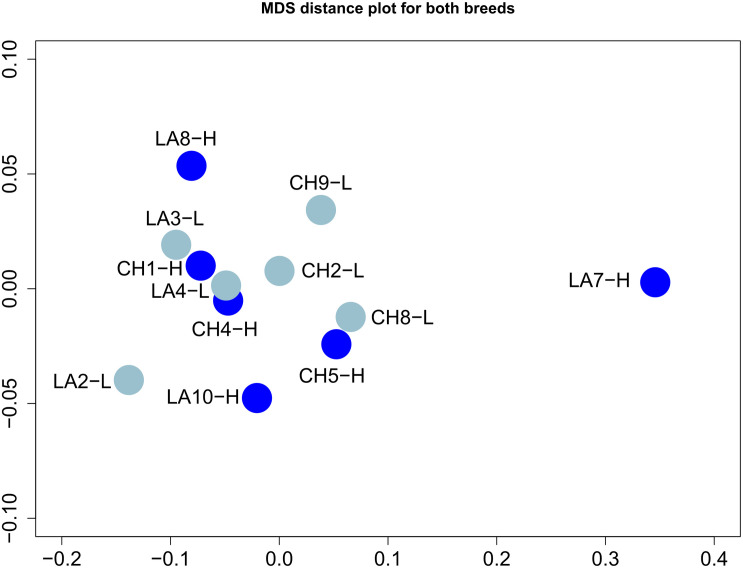
Multidimensional scaling analysis of mammary gland transcriptome profiles using the FPKM (Fragments Per Kilobase Million) values for the 27,327 identified genes. Samples are colored according to their yield (light blue for low yield; blue for high yield).

Distribution of gene expression levels based on FPKM values (presented in log_2_ scale) demonstrated similar profiles for all samples, except for LA7-H (Supplementary Figure 4). The highest FPKM value was observed for samples CH5-H (76,015.58) and LA10-H (57,097.89), both for *PAEP* (Progestagen Associated Endometrial Protein) gene. The level of relatedness of samples based on the FPKM values and the multidimensional scaling analysis of Pearson pairwise correlation coefficient revealed that samples grouped together except for LA7-H, which was found in greater distance compared to the others, confirming the low values of *r*^2^ index in pairwise comparisons (Figure 1). Interestingly, of the 5,761 genes uniquely expressed in Lacaune breed 893 genes were expressed only in LA7-H, demonstrating, however, low FPKM values (median FPKM value = 7.48). Yet, phenotypic measures (milk yield performance, CM T test, BCS) could not justify this differentiation compared to the other Lacaune HY ewes. Overall, no particular correlation among breeds or groups was observed.

### Highly Expressed Genes

**FIGURE 2:**
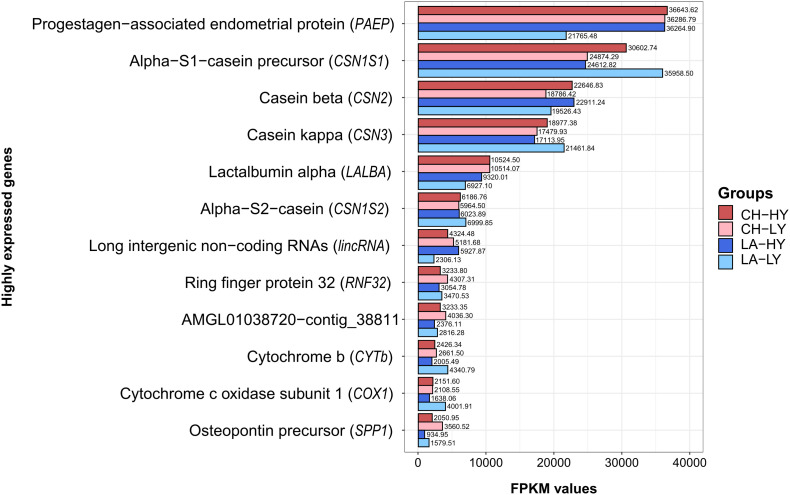
Barplot of the highly expressed genes in milk somatic cells for each breed and group based on the average FMKP (Fragments Per Kilobase Million) values per group. CH, Chios; LA, Lacaune; HY, High yield; LY, Low Yield.

Our results showed that only a small number of genes support the transcriptional activity of the mammary gland after the peak of normalized lactation. Since comparison of top 10 highly expressed genes (HEGs) among the four studied groups showed some discrepancies, gene expression levels for the top HEGs are presented as the average FMKP per group (Figure 2). In our analysis, the top 10 and 30 HEGs accounted for the 32.49 and 39.68% of expressed genes in all groups, respectively. The percentage of top 10 or 30 HEGs in our study was found significantly lower compared to previous studies on the Churra and Assaf sheep, for which the top 10 HEGs accounted for 70% of the expressed genes (Suárez-Vega et al., 2015) or the Romney breed, for which the top 30 HEGs accounted for 60% of all RNA-seq reads (Paten et al., 2015). Since the sequencing depth among studies was similar, these differences can be attributed to the higher number of expressed genes identified in our study and moreover, close to the end of lactation, expression of caseins and lactalbumins starts to drop (Sigl et al., 2012). Consequently, the impact of HEGs on the total activity of the mammary gland can be decreased. In addition, major influence on a gland’s gene expression is the environment, both in terms of the surrounding world in which the organism is found and develops, as well as in terms of an organism’s internal microenvironment, linked to its metabolic activities and endocrine functions (Lobo, 2008).

From the top 10 HEGs within each group, seven genes were common among all groups (Supplementary Figure 5). These genes were associated with caseins *CSN1S1* (alpha S1 casein precursor), *CSN2* (beta casein), *CSN3* (kappa casein), *CSN1S2* (alpha S2 casein) together with *PAEP, LALBA* (lactalbumin alpha) and *lincRNA* (Long intergenic non-coding RNAs). This is expected for milking ruminants and has been previously described for sheep (Paten et al., 2015; Suárez-Vega et al., 2017a,b), cattle (Wickramasinghe et al., 2012; Cánovas et al., 2014; Cui et al., 2014), and goats (Shi et al., 2015; Crisà et al., 2016). Among HEGs, uncharacterized proteins or novel transcripts were encountered that are not fully annotated in Oar_v3.1, hence are presented as scaffolds, contigs or position on chromosomes. Interestingly, sample LA2-L, although a low yield ewe, expressed the highest levels for *CSN1S1* (FPKM = 53,854.17), even when compared to HY ewes [max. FPKM for Lacaune breed (LA-10): 50,316.38, max FPKM for Chios (CH5-H): 47,597.92] (Supplementary Figures 5B,D). In addition, LA8-H expressed the lowest FPKM values for *PAEP* (FPKM = 11,839.44), compared to all ewes included in our study. HEG analysis also revealed that differentiation of LA7-H could be attributed to the increased expression of *lincRNAs* and uncharacterized genomic regions compared to the other ewes studied.

The highest expressed gene for all groups was the *PAEP* gene. This gene, also known as *BLG*, is a major whey protein and encodes for β-lactoglobulin. Similar to previous studies we characterized this gene as a HEG; in previous studies though, its expression levels and ranking among HEGs varied greatly, depending on the stage of sampling (time-point) and breed (Wickramasinghe et al., 2012; Cánovas et al., 2014; Cui et al., 2014; Paten et al., 2015; Shi et al., 2015; Crisà et al., 2016; Suárez-Vega et al., 2017a,b). In cattle, its expression decreases during transition to late lactation, and although it is the major HEG at early lactation (day 15) in Holstein cows, its expression progressively reduces, until it is not found among HEGs at the end of lactation (day 250) (Wickramasinghe et al., 2012). This observation, however, was not corroborated by other studies in sheep (Suárez-Vega et al., 2015) nor by our study; thus, further research could elucidate the variation of *PAEP* expression levels during different lactation and pregnancy stages. In the present study, among Chios and Lacaune breeds, similar FPKM values for *PAEP* gene were obtained for all groups except for LA-LY group, which demonstrated significant lower expression levels. Although β-lactoglobulin is the major whey protein in ruminants, its role and impact on milk phenotype are still controversial (Le Maux et al., 2014; Selvaggi, 2015). Recently though, polymorphisms in β-lactoglobulin gene gained more attention due to their association to milk fat and protein content (Padilla et al., 2018) and milk clotting properties (Suárez-Vega et al., 2017a).

In the present study, significantly higher expression levels were found in the LA-LY group for *CSN1S1* and *CSN3* genes compared to the other groups; FPKM values were 35,958.50 and 21,461.84, respectively. Apart from *CSN1S1*, *CSN3*, and *CSN1S2* expression levels in LA-LY, for the top 6 HEGs, HY groups presented increased FPKM values compared to LY groups. However, for the remaining HEGs the opposite was true, with LY groups expressing higher FPKM values compared to the respective HY groups. The genes that complete the list of HEGs in our study include *lincRNA* ENSOARG00000025951, *RNF32* (Ring finger protein 32), *CYTb* (Cytochrome b), *COX1* (Cytochrome c oxidase subunit 1), and *SPP1* (Secreted phosphoprotein 1). Although *COX1* gene was not among the top 10 of any comparison studied, its high values of FPKM in Chios groups (ranked as 11th HEG with mean FKPM values 2,151.60 and 2,108.55 for CH-HY and CH-LY, respectively), placed this gene among the HEGs of all comparisons. Moreover, contigs JH922760 (541:4784) and AMGL01014165-contig_14204, are not placed among the overall top HEGs, due to the low mean FPKM values compared to all other groups. Interestingly, for the aforementioned contigs, large differences in expression levels between LA-HY and LA-LY groups were observed.

Following the genes encoding for milk proteins, expression of *lincRNA* ENSOARG00000025951 and *RNF32* were the most abundant in terms of normalized FPKM values. ENSOARG00000025951 presented similar FPKM values among groups (5,181.68 (CH-LY), 4,324.48 (CH-HY), 5,927.87 (LA-HY), and 2,306.13 (LA-LY). Overall, in our analysis 74 unique *lincRNAs* were identified; *lincRNAs* are long (>200 nt) non-coding RNAs located in intergenic regions (Shore et al., 2012). They are dispersed throughout the genome and their role has been monitored in several species, trying to elucidate their contribution to cell differentiation, transcriptional control and epigenetic mechanisms. In ruminants, numerous *lincRNAs* have been found in QTLs affecting traits like milk quality and yield or clinical mastitis (Tong et al., 2017); yet, their exact role in mammary gland during lactation remains to be explored. The role of *RNF32* is also unclear; ring finger proteins are involved in the ubiquitin system or can contribute to apoptosis (Joazeiro and Weissman, 2000). Genes that complete the list of HEGs apart from caseins and whey proteins seem to support indirectly mammary gland’s activity by regulating the expression of other genes. For example, *SPP1* gene encoding for osteopontin protein, is a major phosphoglycoprotein highly expressed in the mammary gland, previously associated with induction of casein expression (Sheehy et al., 2009).

### Differentially Expressed Genes

For the investigation of the biological insights that could be highlighted by RNA-seq analysis, a list of DEGs that differentiate the different groups was acquired. The highest number of DEGs was obtained for CH-LY and LA-LY pairwise comparison (*N* = 2,092), whereas the lowest was acquired when comparing high yield ewes between breeds (CH-HY and LA-HY groups) (*N* = 227).

**FIGURE 3:**
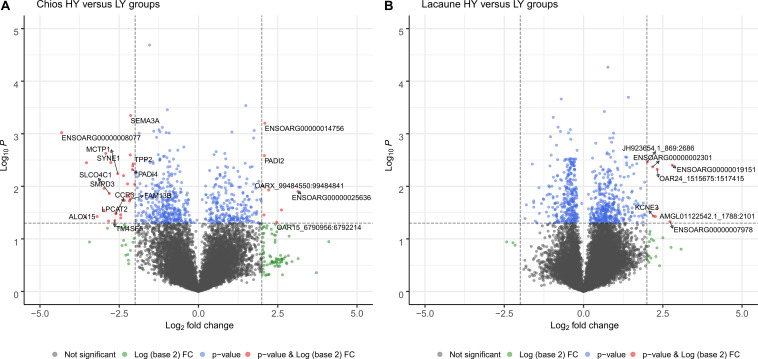
Distribution of genes as a volcano plot according to level of significance for **(A)** Chios and **(B)** Lacaune breeds. Horizontal dashed lines signal the threshold for statistical significance, presented in log_10_ scale (differentially expressed genes with *p*-value < 0.05). Values in *x*-axis represent the level of differentiation of genes (log_2_fc). Dashed vertical lines set the limits outside of which expression changes by fourfold; genes (marked in red) with log_2_fc < –2 and *p*-val < 0.05 represent genes that are upregulated in HY groups and genes with log_2_fc > 2 and *p*-val < 0.05 represent genes that are overexpressed in LY groups.

For Chios breed (HY vs. LY) after filtering out transcripts with a sum of FPKM < 1, 21,566 transcripts remained to be further evaluated in differential expression analysis. Six-hundred and five genes with *P* < 0.05 were found to be differentially expressed; 371 genes were found to be upregulated in CH-HY (fc < 1 and log_2_fc < 0), and 234 genes were upregulated in CH-LY (fc > 1 and log_2_fc > 0). Of them, 37 genes were differentially expressed by fourfold; 29 genes were upregulated in CH-HY and 8 genes were upregulated in CH-LY (Figure 3A and Supplementary Table 4). Among DEGs, *ENSOARG00000008077* was upregulated with the greatest level of differentiation in CH-HY. It is a structural constituent of ribosomes and belongs to the universal ribosomal protein 14 family. Ribosomal proteins are commonly found among the highly expressed genes in the mammary gland. In sheep, Wang et al. (2020) found that ribosomal protein S29 (*RPS29)* is highly expressed in both lactating and non-lactating sheep, while Paten et al. (2015) found that ribosomal protein genes are highly expressed during late pregnancy compared to early lactation. Ribosomal protein genes have also been identified as DEGs elsewhere; in cattle many ribosomal protein genes were downregulated in cows with high milk protein and fat percentage (Cui et al., 2014) and members of this gene family were found to be overexpressed in ewes uninfected from *Mycoplasma agalactiae* (Chopra-Dewasthaly et al., 2017). Overexpression of such proteins has also been reported in other tissues such as at the corpus luteum of high prolific sheep (Pokharel et al., 2020). Overexpression of genes belonging to the ribosomal protein family is biologically meaningful due to their essential role in cellular processes hence, in our data, the transcriptional activity of mammary gland in Chios HY group is probably increased to support the high levels of milk synthesis. Other genes that are upregulated in CH-HY are *LPCAT2* (Lysophosphatidylcholine acyltransferase 2), *CCR3* (C-C chemokine receptor type 3), *MCTP1* (Multiple C2 and transmembrane domain containing 1), *CSTA* (Cystatin A), *KIAA0100* (KIAA0100 ortholog), and *AHR* (Aryl hydrocarbon receptor). In LY group, DEGs mostly referred to uncharacterized proteins or novel transcripts that are not fully annotated in Oar_v3.1. Among the annotated genes that are upregulated in LY Chios ewes is *ENSOARG00000025636* belonging to *lincRNAs* and *PADI2* (Peptidyl arginine deiminase 2) which is involved in calcium ion and estrogen receptor binding. Although its role in sheep is still unclear, *PADI2* was found to be involved in lipid metabolic process in sows (GO:0006629) (Østrup et al., 2010). Further research is required to clarify the enriched lipid transfer activity in low yield ewes, since similar results were reported by Dai et al. (2018) for cows fed with low quality forage, favoring the idea that the low acetate supply from feed triggers the increased uptake of fatty acids from blood for metabolism in the mammary gland (Dai et al., 2018). Overall, comparison of groups within Chios breed revealed that genes upregulated in HY group are enriched in ribosomes, coordinating for milk synthesis, suggesting that the involved pathways might play a managing role in increasing milk yields of the mammary gland.

Within the Lacaune breed (HY vs. LY), 680 transcripts were differentially expressed between groups; 298 genes were upregulated in LA-HY and 382 genes were upregulated in LA-LY. However, only eight genes were upregulated by fourfold, all in LA-LY group (Figure 3B and Supplementary Table 5). These genes were not linked to milk production, but were either related to immune response (ENSOARG00000002301) or involved in the differentiation and survival of nerve cells in sheep such as *KCNE3* (Ma et al., 2019). This result could be an indicator of the health status of these ewes. The Lacaune LY ewes were probably misclassified as healthy animals, yet, results revealed that the reduced productivity of this group might be due to underlying disease-related factors that lurk, but not yet detected or expressed. In fact, through transcriptome sequencing in phenotypically healthy ewes, we have found that *Streptococcus* species and parasites of the Babesia genus are present in the mammary gland of another Greek sheep breed, the Frizarta, thereby adversely affecting milk performance of the ewes (unpublished data). These data support and highlight the need to focus on flock health and improve hygiene conditions in farms, to permit ewes express fully their genetic potential.

Comparison of high yielding ewes between breeds resulted in 27,294 genes; 227 genes were found to be differentially expressed among HY groups. Of them, nine and six genes were upregulated by fourfold in CH-HY and LA-HY groups, respectively (Supplementary Figure 6 and Supplementary Table 6). Again, uncharacterized proteins (ENSOARG00000002701) and *lincRNA* ENSOARG00000025556 were among DEGs. *GPSM2* gene was also found to be upregulated in CH-HY; recently, *GPSM2* has been identified as a DEG, downregulated in Murciano-Granadina dairy goats subjected to heat stress conditions (Contreras-Jodar et al., 2018). Moreover, although *ZNF131* gene is among the most stable expressed genes in ruminant’s endometrium (Walker et al., 2009) in our analysis it was identified among DEGs upregulated in Chios HY ewes compared to Lacaune HY group. This gene has also been identified as a nearby gene to a SNP affecting lactation persistency in Holstein cows (Do et al., 2017). Taking into account that Chios ewes were close to the end of the normalized lactation, this gene, in addition to the well-established QTL in OAR11 (13.6–38.1 Mbp) (Jonas et al., 2011), could be a candidate marker for lactation persistency that needs to be further evaluated. Interestingly, *ASIP* locus was overexpressed in LA-HY and although this locus is connected to coat color pigmentation antagonizing the melanocortin-1 receptor (MC1R) in many farm animals (Fontanesi et al., 2010, 2011; Mao et al., 2010), its role in fat-related traits due to its expression in adipocytes has also been proposed in livestock (Yabuuchi et al., 2010; Albrecht et al., 2012). Yet, since milk fat content in ruminants is driven by both biological (genetic factors, lactation stage, breed, health status) and external (environmental and management conditions, nutrition) factors (Linn, 1988; Hanus et al., 2018), structured multifactorial experiments must be designed before characterizing this gene as a biomarker for milk fat.

Comparison of LY ewes between breeds resulted in many DEGs with *P*-value < 0.05 (*N* = 2,092), pointing to a differentiated expression profile between groups. Of them, 223 genes were up or down regulated by fourfold; 168 and 55 genes were upregulated for LA-LY and CH-LY, respectively (Supplementary Figure 7 and Supplementary Table 7). At more stringent criteria though (*p* < 0.01), this DEGs were reduced to 16. A high percentage of the annotated DEGs overexpressed in CH-LY was directly related to ferritin (Fe) and iron homeostasis (27.7%) and expression of beta defensins (11.1%). To our surprise, *FTH1* (ferritin heavy chain 1), was identified among DEGs, also upregulated in CH-LY. Since it encodes for a major intracellular iron-storage protein, highly expressed during lactation in cattle (Wickramasinghe et al., 2012) and goats (Crisà et al., 2016), we would expect not be encountered among DEGs. In fact, Wickramasinghe et al. (2012) support the idea that *FTH* might play a protective role during peak, late lactation and involution. Iron is essential to living organisms serving as electron donor and acceptor, yet, excessive iron is harmful causing oxidative damage to cells (Li et al., 2020). It is considered that high iron levels lead to efficient translation of ferritin mRNA, favoring iron sequestration over uptake (Cairo and Recalcati, 2007). Also, iron and zinc levels in blood have been previously described as indicators of immune function, correlating their low levels to a non-specific host-defense mechanism against bacterial infection (Miglio et al., 2018). Hence, low ferritin levels in LA-LY ewes could be interpreted as a health indicator and co-evaluated with known factors that influence serum trace mineral concentrations such as nutrition, and seasonal/physiologic variations such as gestation and lactation stages (Herdt and Hoff, 2011). β-defensins, also described by the term host defense peptides, constitute important components of the innate immune system. Until recently 43 β-defensin genes have been characterized for sheep, of them *oBD1* or *SBD1* and *oBD2* or *SBD2* have been thoroughly studied, whereas the remaining are not yet fully annotated to OAR v4.0 (Hall et al., 2017). In bovine species though, expression of β-defensins is much higher in tissues derived from udders infected by bacteria compared to bacteria-free udders (Kościuczuk et al., 2014). Another gene upregulated in Chios breed, *EIF4EBP1* (eukaryotic translation initiation factor 4E binding protein 1), was previously identified among DEGS upregulated in mastitis resistant compared to susceptible cows (Bonnefont et al., 2011). DEGs upregulated in LA-LY ewes revealed a group of key genes related to inflammation or regulation of immune responses like a cluster of interleukins enclosing *IL12RB1*, *IL1RL1*, *IL2RA*, *IL2RA*, and *IL7R*. Overall, the identified group of genes upregulated in CH-LY compared to LA-LY support a putative role in the innate defense of sheep mammary gland, rendering them interesting targets to diagnose or treat various underlying health conditions.

### Annotation and Gene Ontology of the Differentially Expressed Genes

Pathway and function enrichment analyses of DEGs against Oar_v3.1 genome classified genes into three categories: biological process, cellular component, and molecular function. Since ShinyGO did not recognize many of the DEGs, functional annotation was based on a lower number of genes. Overall, 683 unique biological process terms (GO-BP), 155 cellular components (GO-CC) and 153 molecular functions (GO-MF) were enriched at FDR < 0.05.

**FIGURE 4:**
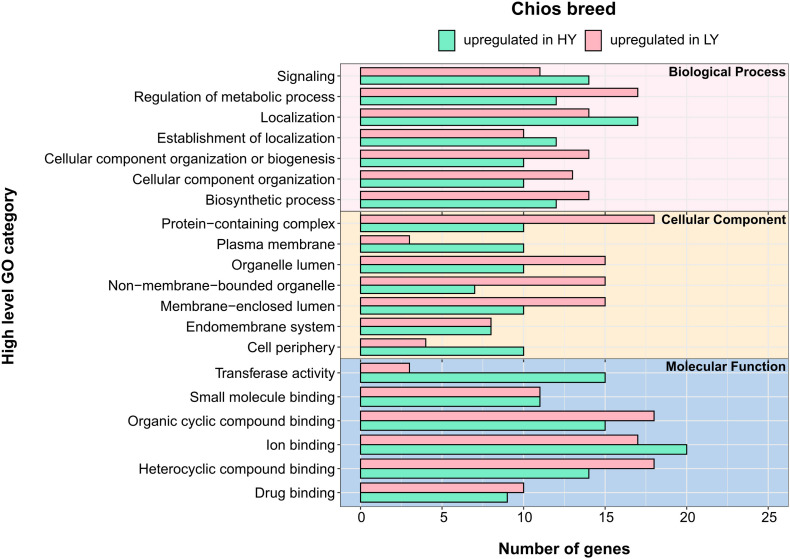
Annotation of differentially expressed genes (DEGs) for the three categories in Chios breed: biological process, cellular component and molecular function. *Y*-axis illustrates functional categories after annotation of DEGs. *X*-axis indicates the total number of genes involved in each functional category. Green bars indicate terms that are upregulate in high yielding animals (HY) whereas pink bars indicate the terms that are upregulate in low yielding animals (LY).

**FIGURE 5:**
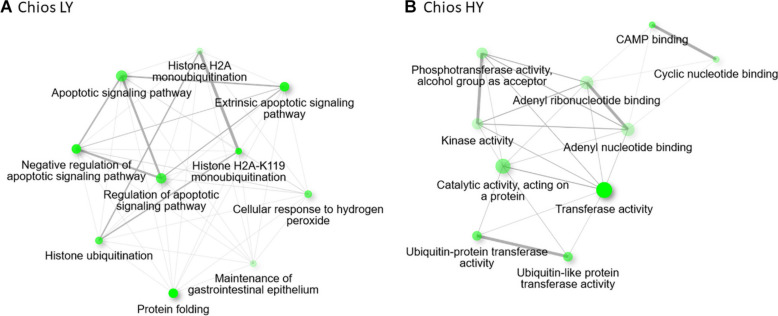
Enriched gene ontology terms based on the annotated differentially expressed genes, visualized as a network, for **(A)** Chios low yielding group and **(B)** Chios high yield animals.

Within Chios breed, DEGs were annotated in 63 GO-BP, 28 GO-CC, and 17 GO-MF unique categories. In Figure 4 the top five GO categories within each group are presented, thus, the total number of terms is increased. Gene annotation of DEGs revealed similar enriched pathways among HY and LY groups. Yet, GO enrichment analysis revealed that gene sets upregulated in Chios LY group, are enriched in histone ubiquitination (GO:0036353, GO:0016574, GO:0035518), apoptotic signaling pathway (GO:2001234, GO:0097191, GO:2001233, GO:0097190), response to hydrogen peroxide (GO:0042542, GO:0070301), protein folding (GO:0006457), and maintenance of gastrointestinal epithelium (GO:0030277) (Figure 5A). Genes involved in the clusters of the enriched GO terms, especially those related to histone ubiquitination and apoptosis, were probably triggered and expressed as response to environmental factors such as heat, osmotic, oxidative or heavy metal stress (Flick and Kaiser, 2012). In fact, Flick and Kaiser (2012), described that core components of the ubiquitin system can function as stress sensors leading in low productivity, hence, performances of CH-LY ewes could be attributed to their reduced resilience against extreme environmental factors during sampling (for example high temperatures in the summer), compared to Chios high yielding ewes. Since heat stress downregulates the expression of caseins (particularly *CSN1S1* gene expression) (Hu Z. L. et al., 2016; Yue et al., 2020) this hypothesis should be further investigated considering the differences in *CSN1S1* expression levels between CH-HY and CH-LY groups. For CH-HY group, pathways that were enriched included categories in molecular function such as transferase activity (GO:0016740, GO:0004842, GO:0019787), CAMP binding (GO:0030552), cyclic nucleotide binding (GO:0030551), tyrosine kinase binding (GO:0030971, GO:1990782), lipid transfer activity (GO:0005319), and ATP binding (GO:0005524) (Figure 5B), suggesting an inherent increased activity of the mammary gland compared to the low yield ewes. In fact, comparative transcriptome analysis of the mammary gland in cows fed with different diets (alfalfa hay vs. corn stover), revealed decreased expression of several ATPase components for cattle fed with low-quality forage, verifying that oxidative phosphorylation that takes place in mitochondria involving several ATP pathways to generate energy are crucial for increased milk production (Dai et al., 2018).

**FIGURE 6:**
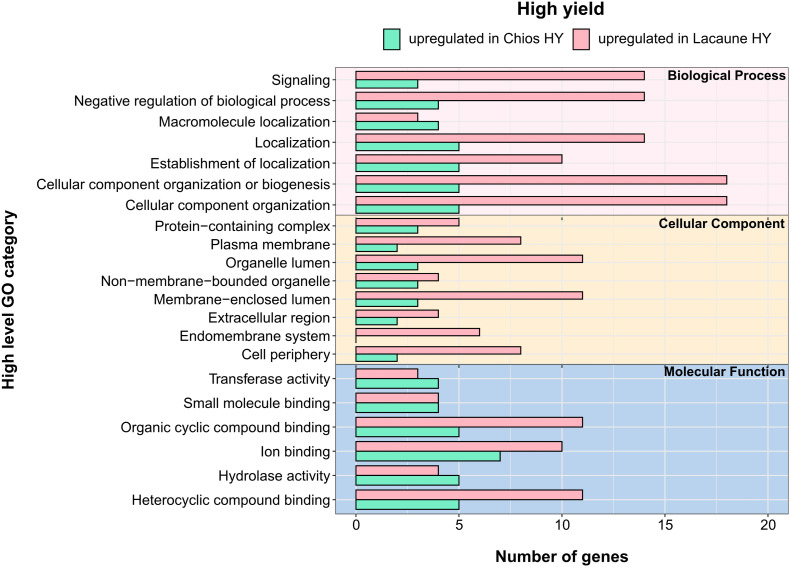
Annotation of differentially expressed genes (DEGs) for the three categories in high yield groups: biological process, cellular component and molecular function. *Y*-axis illustrates functional categories after annotation of DEGs. *X*-axis indicates the total number of genes involved in each functional category. Green bars indicate terms that are upregulate in Chios high yielding animals (CH-HY) whereas pink bars indicate the terms that are upregulate in Lacaune high yielding animals (LA-HY).

We further evaluated the acquired DEGs between the high yielding ewes of both breeds to point out presumable differences in the functional clustering of genes that set up the gland’s transcriptional activity. Comparison of HY groups across breeds revealed differences for Chios and Lacaune high yielding ewes. In total, DEGs were annotated in 51 GO-BP, 23 GO-CC, and 14 GO-MF unique categories. Independently of the category studied, all but one (endomembrane system) pathways that were detected in Chios breed, were also found in Lacaune breed. Moreover, annotated genes for Lacaune HY ewes were categorized in more groups (GO-BP: 20 in Chios vs. 51 in Lacaune, GO-CC: 9 in Chios vs. 22 in Lacaune, GO-MF: 8 in Chios vs. 14 in Lacaune), accompanied by a higher number of genes involved within each category, compared to Chios HY ewes (Figure 6). However, GO enrichment analysis revealed only two significant enriched functional categories upregulated in Chios HY ewes, both related to response to zinc ion (GO:0071294, GO:0010043). Although the importance of Zn in livestock nutrition has been well-established (Hill and Shannon, 2019), its fundamental role in catalytic, structural and regulatory functions in proteins impact virtually all aspects of cell biology (Maret, 2017). Hence, stimulation of zinc ion response in the mammary gland of high yielding ewes needs to be further interpreted with future targeted research in mammalian cells.

For LY ewes, the number of groups, pathways and enriched ontologies was higher compared to the aforementioned comparisons. In particular, DEGs were annotated in 103 GO-BP, 64 GO-CC, and 40 GO-MF unique categories (Supplementary Figure 8). Several enriched ontologies were identified; 140 for Chios-LY and 500 for Lacaune-LY groups. Of them, eight functional GO-BP terms were in common, mostly involving cellular and protein metabolic processes (GO:0051246, GO:0051247, GO:0032268, GO:0044267, GO:0032270, GO:0019538). In CH-LY the most enriched GO categories involved NADH dehydrogenase activity (GO:0010257, GO:0003954, GO:0008137, GO:0050136, GO:0016651, GO:0016651), complexes and functions in mitochondrion (GO:0098798, GO:0005743, GO:0098800, GO:0019866, GO:0005739, GO:0005746, GO:0005740, GO:0031966, GO:0032981, GO:0033108, GO:0007005), protein catabolic processes (GO:0030163, GO:0044257, GO:0042176, GO:1903362), and folding (GO:0006457), mRNA processing (GO:0006397, GO:0045292, GO:0045292) and other functional categories, presented in Supplementary Figure 9A. For LA-LY group, genes involved in the transcriptional regulation of the mammary gland provoked immune response since the most enriched GO pathways included processes in the development or functioning of the immune system, that being lymphocyte activation and differentiation (GO:0046649, GO:0030098, GO:0051249), T cell activation and differentiation (GO:0071610, GO:0033077, GO:0071652), leukocyte activation and differentiation (GO:0045321, GO:0002694, GO:0002521) and immune system processes (GO:0002376, GO:0075528, GO:0002520) (Supplementary Figure 9B).

Similar results to LY groups were obtained within Lacaune breed. Although several groups of genes were annotated to GO-BP, GO-CC, and GO-MF categories (Supplementary Figure 10), GO enrichment analysis revealed functional categories only for Lacaune LY groups, related to immune system development and response, similarly to the LY group comparison (Supplementary Figure 11).

Concluding, results reported here on transcriptome sequencing of the mammary gland between groups of ewes of different performance, supported that within a dairy sheep breed, the innate transcriptional activity of the mammary gland and health status set the base for ewe productivity. In particular, construction of gene regulatory networks based on the differentially expressed genes between groups of different performance, unveiled that the low productivity of ewes that appeared healthy may be due to underlying, or not yet expressed, health problems, since important networks related to immune responses were synthesized. Also, between the high yielding groups of Chios and Lacaune breeds, significant differences in gene expression levels related to zinc response and metabolism were identified, possibly related to nutritional aspects. Our results associate the reduced productivity of two intensively reared dairy sheep breeds with immune defense progressing within the mammary gland. This is a key-point that breeders need to target and control, which will reinforce ongoing breeding programs and provide a shield against future diseases or environmental threats. Although all the identified genes involved in the constructed networks and pathways require further in-depth investigation, the results of this study contribute to the elucidation of the true dynamics of the mammary gland of dairy ewes and can be further exploited by breeders, suggesting to embed preventive veterinary strategies to improve health status of their flock. Overall, the constructed regulatory networks showed that ewe selection based solely on breed to gain maximum profit in terms of milk production is not a *panacea* for a sustainable and profitable dairy sheep farming.

## Data Availability Statement

The datasets presented in this study can be found in online repositories. Sequences have been deposited to NCBI Sequence Read Archive (SRA) under the BioProject PRJNA724691. The links to accession numbers can be found below: https://www.ncbi.nlm.nih.gov/biosample/SAMN18848576; https://www.ncbi.nlm.nih.gov/biosample/SAMN18848577; https://www.ncbi.nlm.nih.gov/biosample/SAMN18848578; https://www.ncbi.nlm.nih.gov/biosample/SAMN18848579; https://www.ncbi.nlm.nih.gov/biosample/SAMN18848580; https://www.ncbi.nlm.nih.gov/biosample/SAMN18848581; https://www.ncbi.nlm.nih.gov/biosample/SAMN18848582; https://www.ncbi.nlm.nih.gov/biosample/SAMN18848583; https://www.ncbi.nlm.nih.gov/biosample/SAMN18848584; https://www.ncbi.nlm.nih.gov/biosample/SAMN18848585; https://www.ncbi.nlm.nih.gov/biosample/SAMN18848586; and https://www.ncbi.nlm.nih.gov/biosample/SAMN18848587.

## Ethics Statement

Ethical review and approval was not required for the animal study because milk from ewes involved in this research was obtained through the daily milking process. Written informed consent was obtained from the owners for the participation of their animals in this study.

## Author Contributions

SM and AA conceived the study. SM, AG, GA, and AA designed the study. SM, AG, and AA performed the sampling. SM performed the interpretation of data and experiment, analyzed the data, and wrote the manuscript. AA, GA, and GB supervised the research. AA acquired the funding. SM, AG, GB, GA, and AA revised the manuscript. All authors contributed to the article and approved the submitted version.

## Conflict of Interest

The authors declare that the research was conducted in the absence of any commercial or financial relationships that could be construed as a potential conflict of interest.

## Footnotes

1 http://ftp.ensembl.org/pub/release-98/fasta/ovis_aries/
