# Supplementary material for: Comparative Transcriptome Analysis of Milk Somatic Cells During Lactation Between Two Intensively Reared Dairy Sheep Breeds
Source: Front Genet. 2021 Jul 19;12:700489. doi: 10.3389/fgene.2021.700489 (PMC8326974; doi:10.3389/fgene.2021.700489)
Supplement: Supplementary file 1 [file Data_Sheet_1.docx]

Supplementary Figures


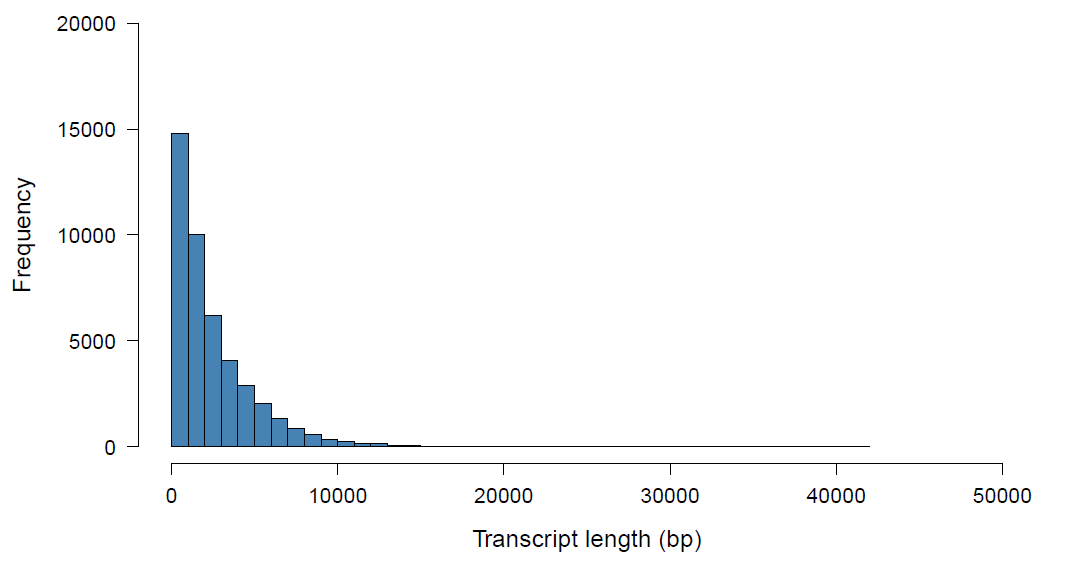


**Supplementary Figure 1.** Distribution of transcript length (bp) and frequency of transcripts for both breeds, after the exclusion of low abundant transcripts.


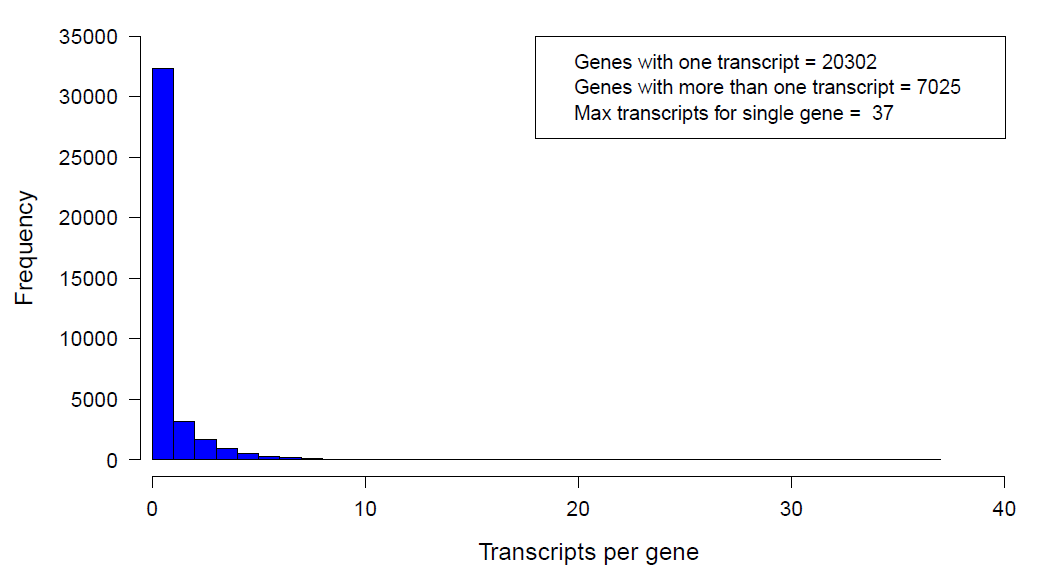


**Supplementary Figure 2.** Distribution of transcript count per gene for both breeds, after the exclusion of low abundant transcripts.


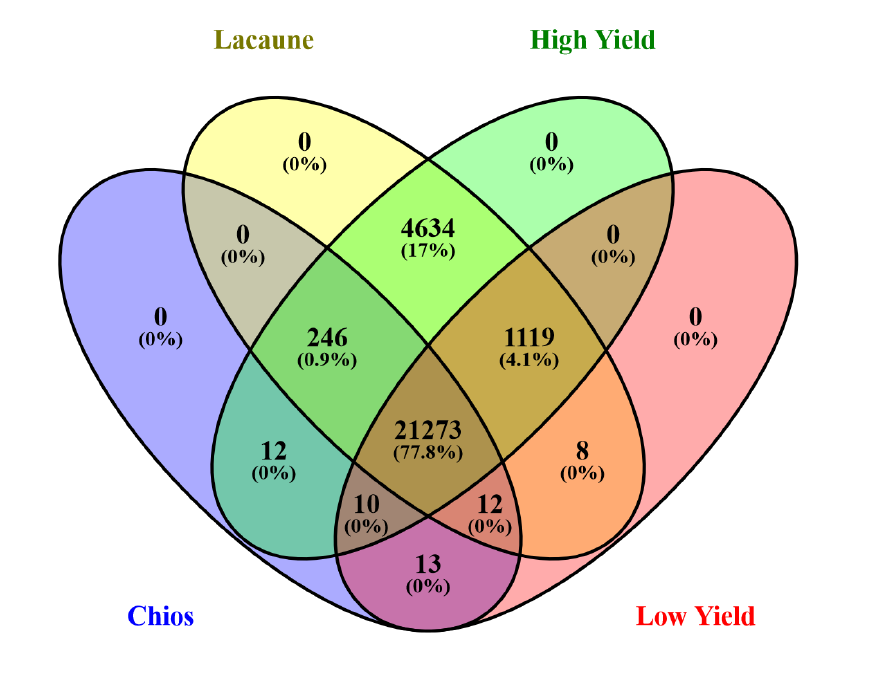


**Supplementary Figure 3.** Venn’s diagrams indicating the number and percentages of unique and shared genes between groups.


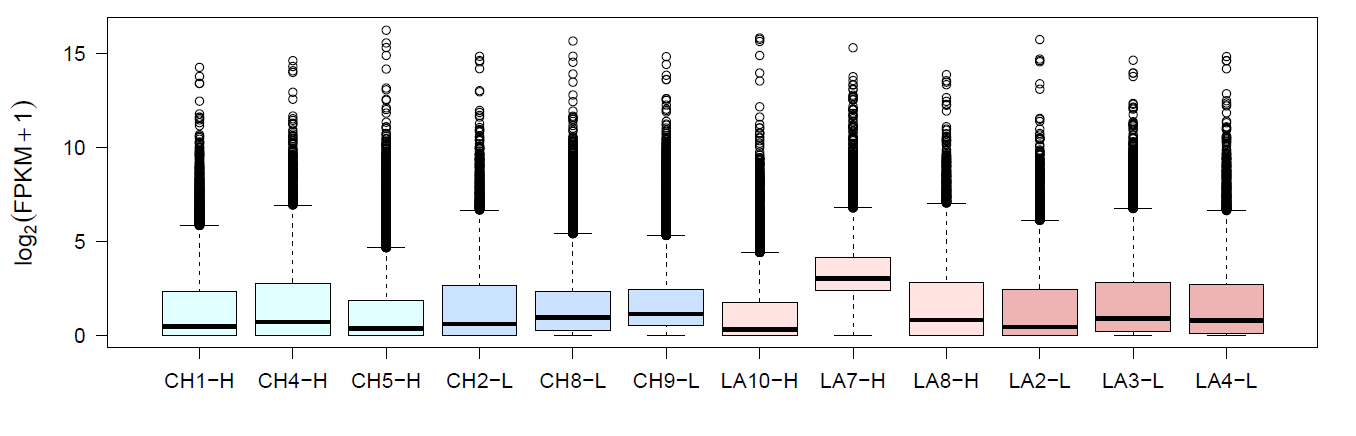


**Supplementary Figure 4.** Boxplots presenting the distribution of FPKM (Fragments Per Kilobase Million) values for both breeds and groups, in a log_2_(FPKM+1) format. Median values are presented with a black and bold horizontal line, whereas extreme values are presented with circles.


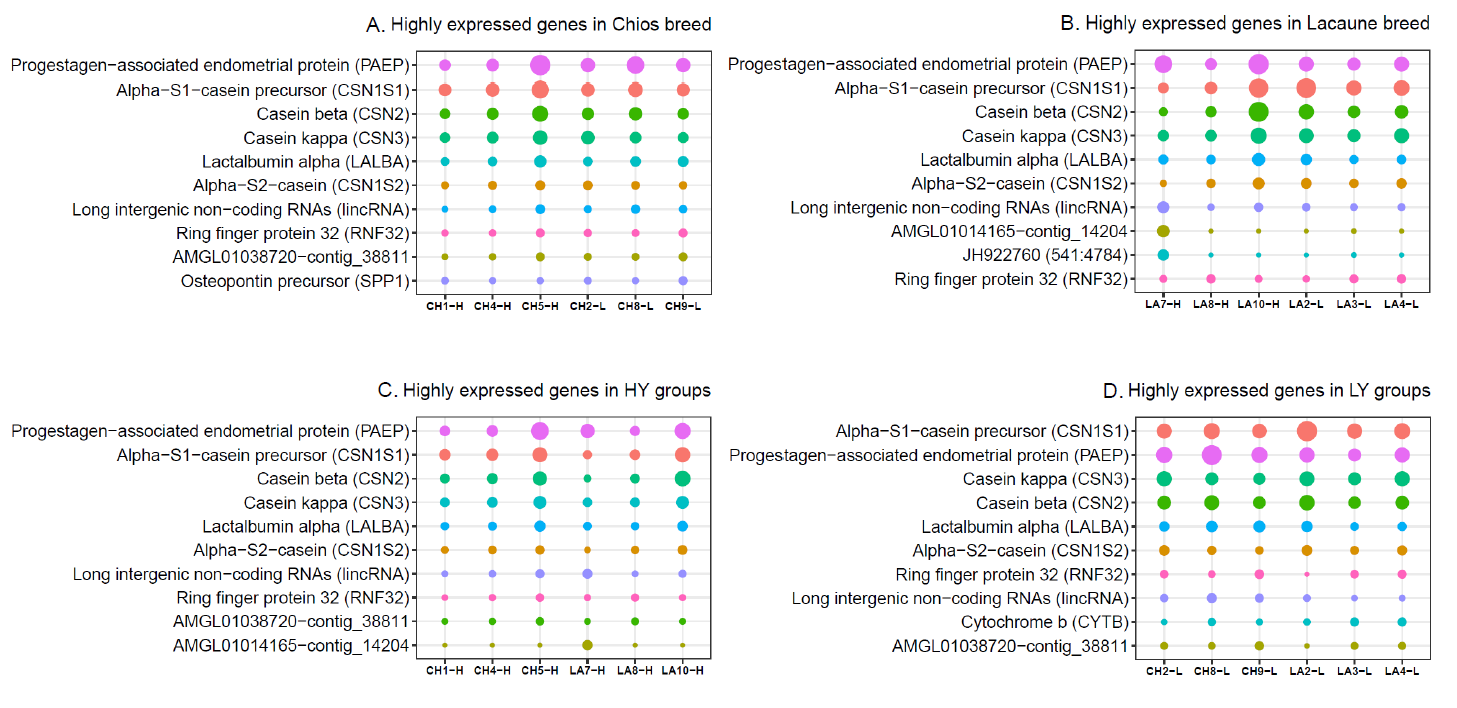


**Supplementary Figure 5.** Highly expressed genes (top 10) for A. Chios, B. Lacaune, C. High yield and D. Low yield groups.


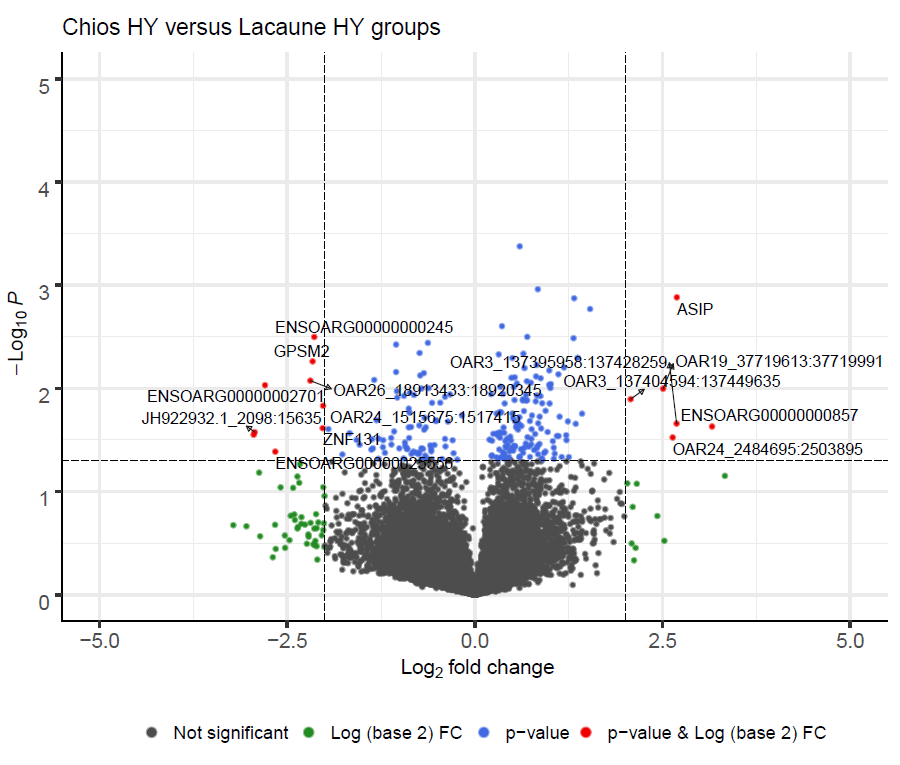


**Supplementary Figure 6.** Distribution of genes as a volcano plot according to their level of significance for Chios high yield (CH-HY) versus Lacaune high yield (LA-HY) groups.


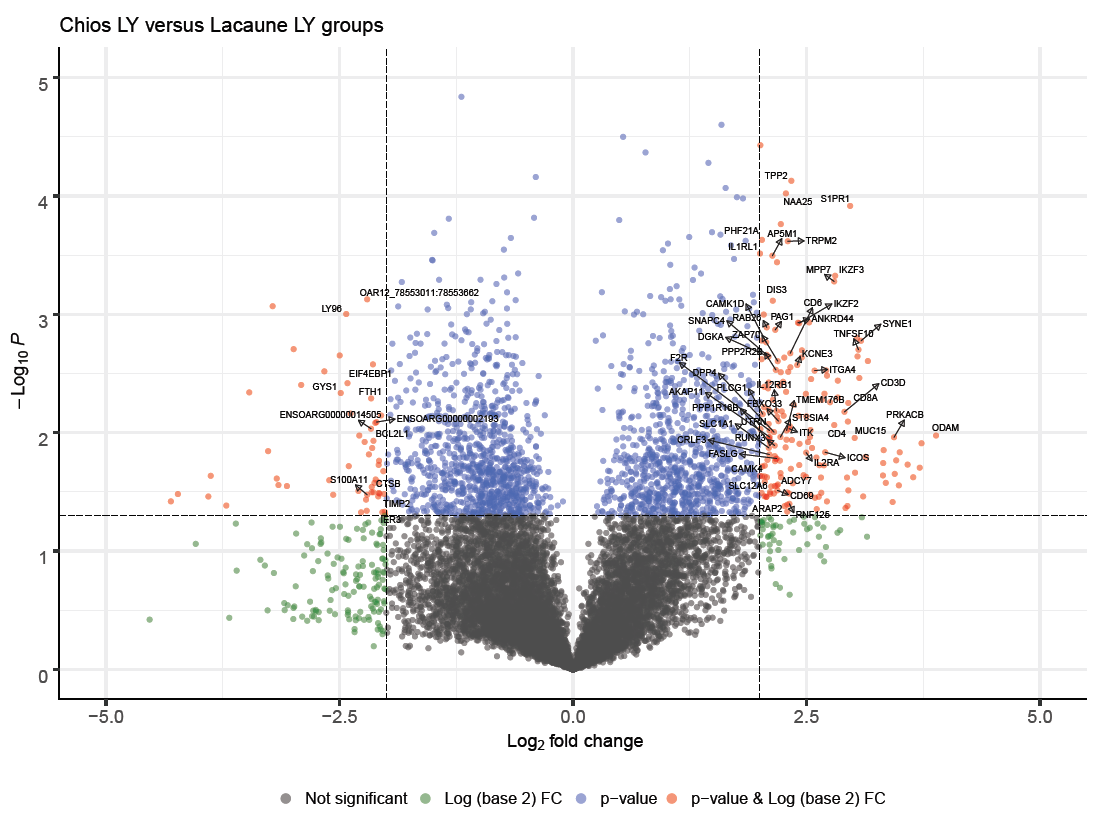


**Supplementary Figure 7.** Distribution of genes as a volcano plot according to their level of significance for Chios low yield (CH-LY) versus Lacaune low yield (LA-LY) groups.


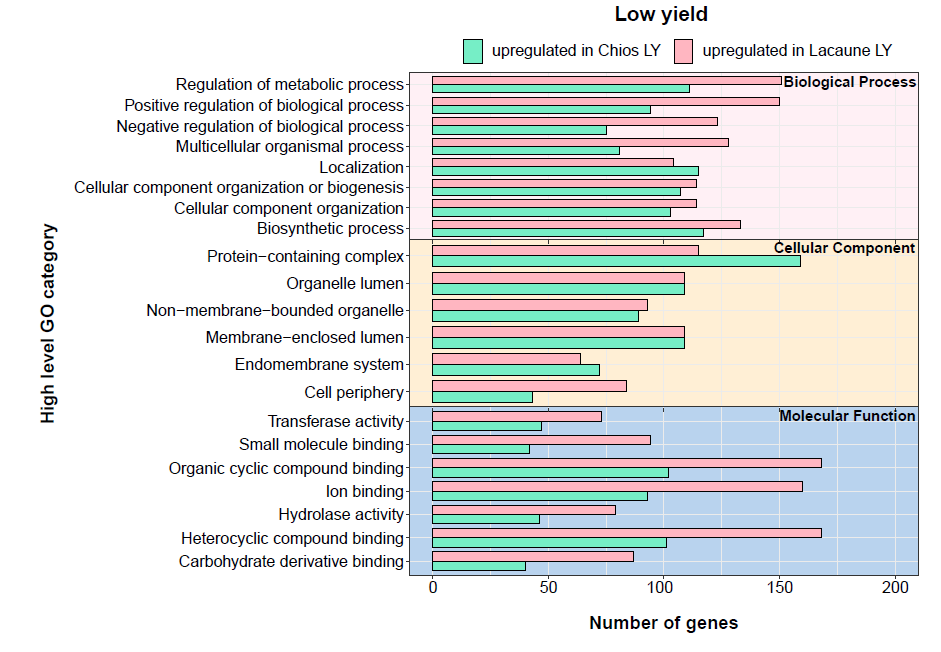


**Supplementary Figure 8.** Annotation of differentially expressed genes (DEGs) for the three gene ontology categories in low yield groups.


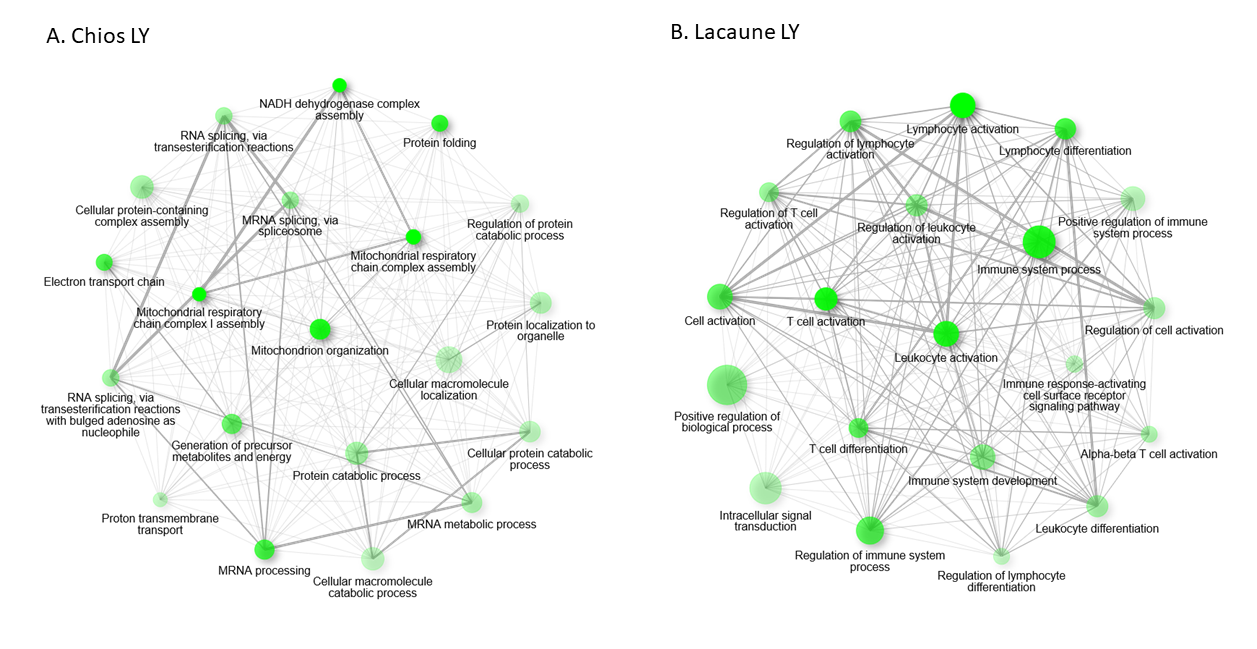


**Supplementary Figure 9.** Enriched gene ontology terms based on the annotated differentially expressed genes visualized as a network, for: A. Chios low yield (CH-LY) group and B. Lacaune low yield (LA-LY) group.


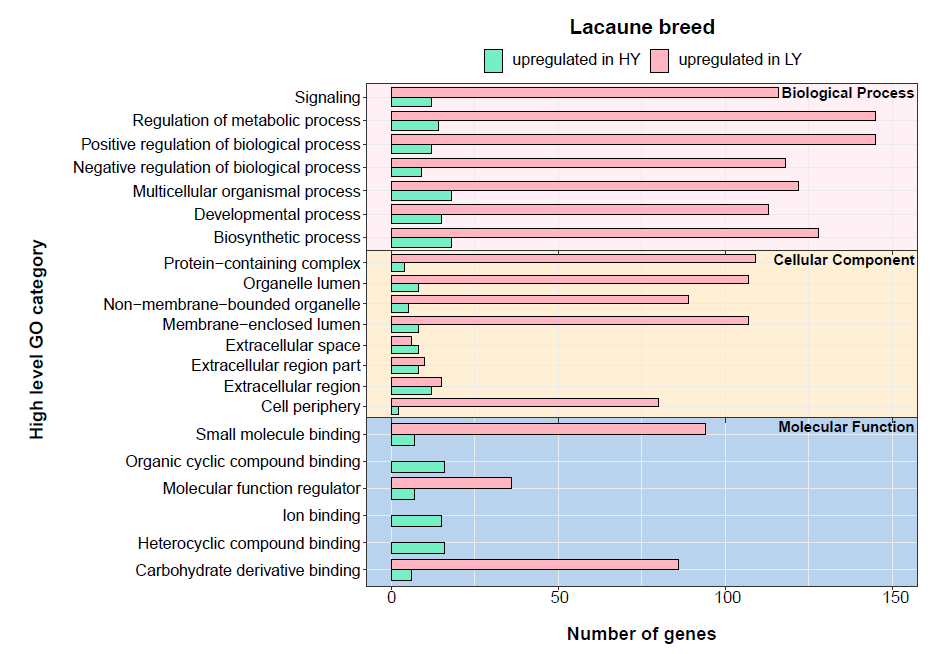


**Supplementary Figure 10.** Annotation of differentially expressed genes (DEGs) for the three gene ontology categories in Lacaune breed.


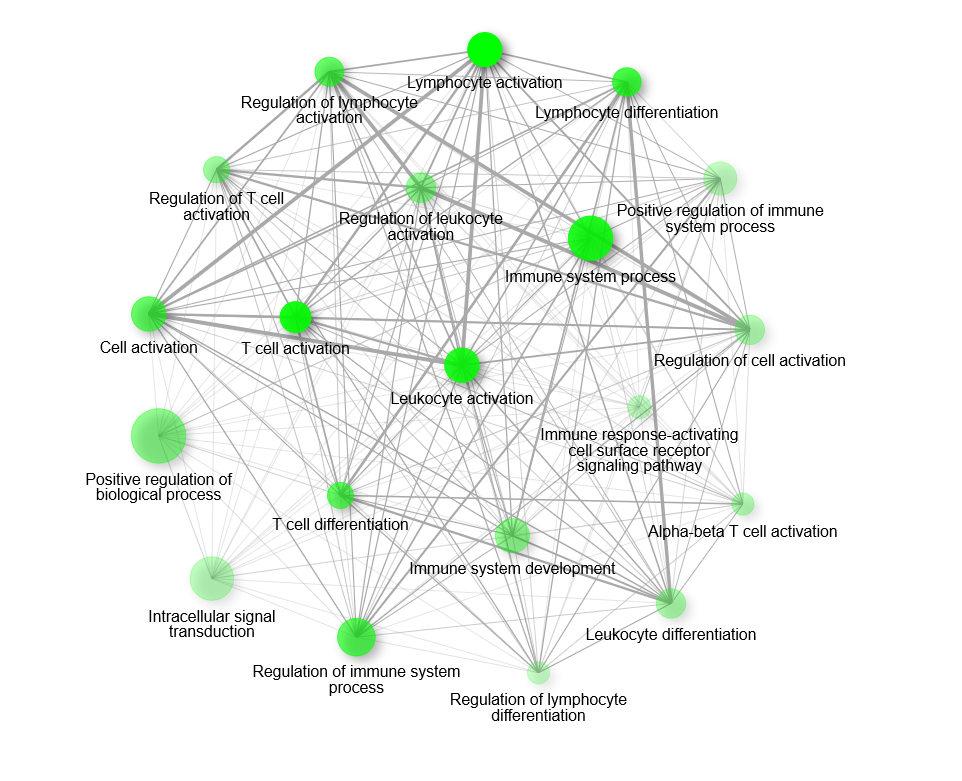


**Supplementary Figure 11.** Enriched gene ontology terms based on the annotated differentially expressed genes visualized as a network for Lacaune low yield (LA-LY) group.
