# Supplementary material for: Comparative Transcriptome Analysis of Milk Somatic Cells During Lactation Between Two Intensively Reared Dairy Sheep Breeds
Source: Front Genet. 2021 Jul 19;12:700489. doi: 10.3389/fgene.2021.700489 (PMC8326974; doi:10.3389/fgene.2021.700489)
Supplement: Supplementary file 2 [file Data_Sheet_2.docx]

Supplementary Tables

**Supplementary Table 1**. Farm locations and values of environmental factors recorded during sampling for each breed. Data were obtained from the Hellenic National Meteorological service (<http://www.hnms.gr/emy/>).

| **Flock breed** | **Latitude** | **Longitude** | **Altitude (m)** | **Temperature (°C)** | | | **Humidity (%)** |
| --- | --- | --- | --- | --- | --- | --- | --- |
|  |  |  |  | **Min** | **Max** | **Average** |  |
| Chios | 39.37896043866203 | 22.86014837042189 | 105 | 16.3 | 29.0 | 24.5 | 53.7 |
| Lacaune | 40.94038419232129 | 22.11835931278313 | 113 | 17.3 | 32.0 | 24.6 | 38.3 |

**Supplementary Table 2**. Summary of sequencing results. Raw and quality-filtered sequences (2 x 75 reads) for each sample. Overall alignment rate for each sample based on the HISAT2 aligner against the Oar_v3.1 reference genome.

| **Sample** | **Raw reads** | **Quality filtered reads** | **Alignment rate** |
| --- | --- | --- | --- |
| **CH1-H** | 27,421,608 | 27,344,106 | 90.71% |
| **CH2-L** | 26,652,226 | 26,431,196 | 87.22% |
| **CH4-H** | 19,544,350 | 19,403,350 | 88.82% |
| **CH5-H** | 24,662,684 | 24,484,326 | 85.81% |
| **CH8-L** | 21,897,324 | 21,673,994 | 85.60% |
| **CH9-L** | 27,531,102 | 27,353,152 | 86.57% |
| **LA10-H** | 25,088,562 | 24,955,626 | 88.93% |
| **LA2-L** | 23,327,492 | 23,235,180 | 89.66% |
| **LA3-L** | 26,164,290 | 26,085,498 | 90.10% |
| **LA4-L** | 34,317,762 | 34,196,160 | 88.91% |
| **LA7-H** | 29,393,260 | 29,225,336 | 84.73% |
| **LA8-H** | 25,936,806 | 25,794,760 | 89.35% |
| **Total** | 311,937,466 | 310,182,684 |  |

**Supplementary Table 3**. Metrics of Pearson correlation coefficient (r^2^) for pairwise comparisons within breeds. Correlations are based on the FPKM (Fragments Per Kilobase Million) values.

|  | **CH1-H** | **CH4-H** | **CH5-H** | **CH2-L** | **CH8-L** | **CH9-L** | **LA10-H** | **LA7-H** | **LA8-H** | **LA2-L** | **LA3-L** | **LA4-L** |
| --- | --- | --- | --- | --- | --- | --- | --- | --- | --- | --- | --- | --- |
| **CH1-H** | 1 |  |  |  |  |  |  |  |  |  |  |  |
| **CH4-H** | 0.92 | 1 |  |  |  |  |  |  |  |  |  |  |
| **CH5-H** | 0.82 | 0.88 | 1 |  |  |  |  |  |  |  |  |  |
| **CH2-L** | 0.89 | 0.95 | 0.91 | 1 |  |  |  |  |  |  |  |  |
| **CH8-L** | 0.82 | 0.86 | 0.98 | 0.88 | 1 |  |  |  |  |  |  |  |
| **CH9-L** | 0.87 | 0.89 | 0.92 | 0.90 | 0.92 | 1 |  |  |  |  |  |  |
| **LA10-H** |  |  |  |  |  |  | 1 |  |  |  |  |  |
| **LA7-H** |  |  |  |  |  |  | 0.41 | 1 |  |  |  |  |
| **LA8-H** |  |  |  |  |  |  | 0.80 | 0.33 | 1 |  |  |  |
| **LA2-L** |  |  |  |  |  |  | 0.86 | 0.27 | 0.81 | 1 |  |  |
| **LA3-L** |  |  |  |  |  |  | 0.82 | 0.32 | 0.90 | 0.91 | 1 |  |
| **LA4-L** |  |  |  |  |  |  | 0.89 | 0.38 | 0.90 | 0.90 | 0.95 | 1 |

**Supplementary Table 4**. Thirty-seven differentially expressed genes for Chios breed. **Transcript ID, Gene ID**: transcript or gene name from www.ensembl.org where available. **fc (Fold change):** Values lower than one in absolute value (fc < 1) declare upregulation in Chios high yielding (HY) animals, values greater than one in absolute value (fc > 1) declare upregulation in Chios low yielding (LY) animals. **pval**: Level of significance. **DE (log_2_fc)**: Negative values declare upregulation in HY whereas positive values declare upregulation in LY.

| **Transcript ID** | **Gene ID** | **fc** | **pval** | **DE (log_2_fc)** |
| --- | --- | --- | --- | --- |
| ENSOART00000008787 | ENSOARG00000008077 | 0.050 | 0.001 | -4.316 |
| JH923118.1_4543:5143 | *uncharacterized* | 0.085 | 0.039 | -3.559 |
| ENSOART00000006433 | ENSOARG00000005914 | 0.086 | 0.004 | -3.532 |
| ENSOART00000009234 | ALOX15 | 0.109 | 0.038 | -3.197 |
| JH922061.1_702:1014 | *uncharacterized* | 0.126 | 0.028 | -2.986 |
| ENSOART00000003716 | SMPD3 | 0.127 | 0.011 | -2.976 |
| ENSOART00000010373 | ENSOARG00000009526 | 0.132 | 0.002 | -2.925 |
| ENSOART00000017447 | ENSOARG00000016020 | 0.140 | 0.046 | -2.837 |
| ENSOART00000019893 | SLCO4C1 | 0.142 | 0.014 | -2.814 |
| ENSOART00000003875 | SYNE1 | 0.147 | 0.004 | -2.763 |
| ENSOART00000007784 | TM4SF5 | 0.161 | 0.045 | -2.639 |
| ENSOART00000019693 | LPCAT2 | 0.164 | 0.033 | -2.604 |
| ENSOART00000015517 | CCR3 | 0.165 | 0.033 | -2.603 |
| ENSOART00000018347 | MCTP1 | 0.171 | 0.006 | -2.546 |
| ENSOART00000005162 | ENSOARG00000004738 | 0.182 | 0.035 | -2.454 |
| JH922978.1_645:1592 | *uncharacterized* | 0.183 | 0.040 | -2.450 |
| ENSOART00000021906 | CSTA | 0.194 | 0.006 | -2.365 |
| OAR3_214166471:214166803 | *NA - uncharacterized* | 0.198 | 0.019 | -2.336 |
| ENSOART00000004117 | TRMT1L | 0.212 | 0.009 | -2.237 |
| OAR2_231610307:231610524 | *uncharacterized* | 0.212 | 0.016 | -2.235 |
| OAR14_35155132:35156753 | *uncharacterized* | 0.221 | 0.019 | -2.176 |
| ENSOART00000017082 | FAM13B | 0.225 | 0.018 | -2.154 |
| OAR2_245624300:245627121 | *uncharacterized* | 0.225 | 0.003 | -2.153 |
| ENSOART00000016636 | SEMA3A | 0.227 | 0.000 | -2.140 |
| ENSOART00000005014 | ENSOARG00000004608 | 0.228 | 0.014 | -2.135 |
| ENSOART00000011986 | PADI4 | 0.236 | 0.005 | -2.083 |
| ENSOART00000001967 | KIAA0100 | 0.238 | 0.004 | -2.073 |
| ENSOART00000005462 | TPP2 | 0.240 | 0.004 | -2.061 |
| ENSOART00000010053 | AHR | 0.248 | 0.009 | -2.014 |
| OAR21_31194401:31194780 | *uncharacterized* | 4.097 | 0.010 | 2.035 |
| OAR7_22534587:22535938 | *uncharacterized* | 4.189 | 0.035 | 2.067 |
| ENSOART00000012128 | PADI2 | 4.230 | 0.003 | 2.081 |
| ENSOART00000016056 | ENSOARG00000014756 | 4.264 | 0.001 | 2.092 |
| OARX_99484550:99484841 | *uncharacterized* | 4.638 | 0.012 | 2.213 |
| OAR15_6790956:6792214 | *uncharacterized* | 5.524 | 0.048 | 2.466 |
| JH922583.1_89:5841 | *uncharacterized* | 6.133 | 0.028 | 2.617 |
| ENSOART00000027573 | ENSOARG00000025636 | 8.800 | 0.013 | 3.137 |

**Supplementary Table 5**. Eight differentially expressed genes overexpressed in Lacaune low yield animals compared to high yield animals. **Transcript ID, Gene ID**: transcript or gene name from www.ensembl.org where available. **fc (Fold change):** Values greater than one in absolute value (fc > 1) declare upregulation in Lacaune low yielding (LY) animals. **pval**: Level of significance. **DE (log_2_fc)**: Positive values declare upregulation in LY.

| **Transcript ID** | **Gene ID** | **fc** | **pval** | **DE (log_2_fc)** |
| --- | --- | --- | --- | --- |
| JH923654.1_869:2686 | *uncharacterized* | 4.031 | 0.003 | 2.011 |
| ENSOART00000020856 | ENSOARG00000019151 | 6.953 | 0.004 | 2.798 |
| ENSOART00000002487 | ENSOARG00000002301 | 4.528 | 0.004 | 2.179 |
| OAR24_1515675:1517415 | *uncharacterized* | 5.002 | 0.005 | 2.322 |
| OAR11_36334797:36346686 | *uncharacterized* | 5.011 | 0.026 | 2.325 |
| ENSOART00000007319 | KCNE3 | 4.614 | 0.037 | 2.206 |
| AMGL01122542.1_1788:2101 | *uncharacterized* | 4.817 | 0.037 | 2.268 |
| ENSOART00000008687 | ENSOARG00000007978 | 6.620 | 0.047 | 2.727 |

**Supplementary Table 6**. Fifteen differentially expressed genes for high yielding (HY) animals among Chios and Lacaune breeds. **Transcript ID, Gene ID**: transcript or gene name from www.ensembl.org where available. **fc (Fold change):** Values lower than one in absolute value (fc < 1) declare upregulation in Chios high yielding (HY) animals, values greater than one in absolute value (fc > 1) declare upregulation in Lacaune high yielding (HY) animals. **pval**: Level of significance. **DE (log_2_fc)**: Negative values declare upregulation in Chios HY whereas positive values declare upregulation in Lacaune HY.

| **Transcript ID** | **Gene ID** | **fc** | **pval** | **DE (log_2_fc)** |
| --- | --- | --- | --- | --- |
| OAR3_213757908:213807644 | *uncharacterized* | 0.130 | 0.028 | -2.946 |
| JH922932.1_2098:15635 | *uncharacterized* | 0.131 | 0.027 | -2.933 |
| ENSOART00000002923 | ENSOARG00000002701 | 0.144 | 0.009 | -2.795 |
| ENSOART00000027486 | ENSOARG00000025556 | 0.158 | 0.041 | -2.659 |
| OAR26_18913433:18920345 | *uncharacterized* | 0.219 | 0.008 | -2.192 |
| ENSOART00000020665 | GPSM2 | 0.224 | 0.005 | -2.161 |
| ENSOART00000000257 | ENSOARG00000000245 | 0.227 | 0.003 | -2.140 |
| ENSOART00000009508 | ZNF131 | 0.245 | 0.024 | -2.027 |
| OAR24_1515675:1517415 | *uncharacterized* | 0.246 | 0.015 | -2.022 |
| OAR3_137404594:137449635 | *uncharacterized* | 4.213 | 0.013 | 2.075 |
| OAR19_37719613:37719991 | *uncharacterized* | 5.685 | 0.010 | 2.507 |
| OAR24_2484695:2503895 | *uncharacterized* | 6.211 | 0.030 | 2.635 |
| OAR3_137395958:137428259 | *uncharacterized* | 6.439 | 0.022 | 2.687 |
| ENSOART00000010326 | ASIP | 6.454 | 0.001 | 2.690 |
| ENSOART00000000913 | ENSOARG00000000857 | 8.932 | 0.023 | 3.159 |

**Supplementary Table 7**. Sixteen differentially expressed genes for low yielding (LY) animals among Chios and Lacaune breeds. **Transcript ID, Gene ID**: transcript or gene name from www.ensembl.org where available. **fc (Fold change):** Values lower than one in absolute value (fc < 1) declare upregulation in Chios low yielding (LY) animals, values greater than one in absolute value (fc > 1) declare upregulation in Lacaune low yielding (LY) animals. **pval**: Level of significance. **DE (log_2_fc)**: Negative values declare upregulation in Chios LY whereas positive values declare upregulation in Lacaune LY.

| **Transcript ID** | **Gene ID** | **fc** | **pval** | **DE (log_2_fc)** |
| --- | --- | --- | --- | --- |
| OAR20_36866327:36866966 | *uncharacterized* | 0.108 | 0.00085 | -3.217 |
| ENSOART00000006931 | LY96 | 0.186 | 0.00099 | -2.429 |
| OAR12_78553011:78553662 | *uncharacterized* | 0.217 | 0.00075 | -2.205 |
| ENSOART00000014253 | IL1RL1 | 4.003 | 0.00031 | 2.001 |
| ENSOART00000006555 | GASK1B | 4.014 | 0.00004 | 2.005 |
| ENSOART00000003288 | PHF21A | 4.061 | 0.00024 | 2.022 |
| ENSOART00000022992 | AP5M1 | 4.388 | 0.00032 | 2.134 |
| ENSOART00000016660 | DIS3 | 4.401 | 0.00077 | 2.138 |
| OAR4_112583580:112658667 | *uncharacterized* | 4.542 | 0.00036 | 2.183 |
| ENSOART00000016052 | *uncharacterized* | 4.672 | 0.00017 | 2.224 |
| ENSOART00000011213 | NAA25 | 4.851 | 0.00010 | 2.278 |
| ENSOART00000013030 | TRPM2 | 4.918 | 0.00024 | 2.298 |
| ENSOART00000005462 | TPP2 | 5.051 | 0.00007 | 2.337 |
| ENSOART00000016801 | MPP7 | 6.940 | 0.00053 | 2.795 |
| ENSOART00000013196 | IKZF3 | 6.991 | 0.00047 | 2.806 |
| ENSOART00000011632 | S1PR1 | 7.814 | 0.00012 | 2.966 |
